# Biological Performance of Duplex PEO + CNT/PCL Coating on AZ31B Mg Alloy for Orthopedic and Dental Applications

**DOI:** 10.3390/jfb14090475

**Published:** 2023-09-16

**Authors:** Morteza Daavari, Masoud Atapour, Marta Mohedano, Endzhe Matykina, Raul Arrabal, Dobrila Nesic

**Affiliations:** 1 Department of Materials Engineering, Isfahan University of Technology, Isfahan 84156-83111, Iran; morteza.daavari@gmail.com; 2 Departamento de Ingeniería Química y de Materiales, Facultad de Ciencias Químicas, Universidad Complutense, 28040 Madrid, Spain; mmohedan@ucm.es (M.M.); e.matykina@quim.ucm.es (E.M.); rarrabal@ucm.es (R.A.); 3 Division of Fixed Prosthodontics and Biomaterials, University Clinic of Dental Medicine, University of Geneva, Rue Michel-Servet 1, CH-1211 Geneva, Switzerland

**Keywords:** biodegradable Mg-based implant, plasma electrolyte oxidation (PEO), multi-walled carbon nanotubes (MWCNT), polycaprolactone (PCL), cell adhesion, cell metabolism

## Abstract

To regulate the degradation rate and improve the surface biocompatibility of the AZ31B magnesium alloy, three different coating systems were produced via plasma electrolytic oxidation (PEO): simple PEO, PEO incorporating multi-walled carbon nanotubes (PEO + CNT), and a duplex coating that included a polycaprolactone top layer (PEO + CNT/PCL). Surfaces were characterized by chemical content, roughness, topography, and wettability. Biological properties analysis included cell metabolism and adhesion. PEO ± CNT resulted in an augmented surface roughness compared with the base material (BM), while PCL deposition produced the smoothest surface. All surfaces had a contact angle below 90°. The exposure of gFib-TERT and bmMSC to culture media collected after 3 or 24 h did not affect their metabolism. A decrease in metabolic activity of 9% and 14% for bmMSC and of 14% and 29% for gFib-TERT was observed after 3 and 7 days, respectively. All cells died after 7 days of exposure to BM and after 15 days of exposure to coated surfaces. Saos-2 and gFib-TERT adhered poorly to BM, in contrast to bmMSC. All cells on PEO anchored into the pores with filopodia, exhibited tiny adhesion protrusions on PEO + CNT, and presented a web-like spreading with lamellipodia on PEO + CNT/PCL. The smooth and homogenous surface of the duplex PEO + CNT/PCL coating decreased magnesium corrosion and led to better biological functionality.

## 1. Introduction

The demand for metallic biomaterials has grown significantly over the past decades due to a constant increase in the aging population, bone diseases, and accidents [1]. Biomaterials developed and used in orthopedics and dentistry for fracture fixation and joint replacement are non-degradable metals, such as titanium, titanium alloys, cobalt-chrome alloys, and stainless steel [2,3]. Despite their excellent mechanical properties and biocompatibility, non-degradable metals present several drawbacks, including stress-shielding effects; undesirable host responses, including inflammation and the release of wear particles; and a necessity for a second removal surgery [4,5]. Among degradable metallic materials, magnesium (Mg) and its alloys have found their place in orthopedic and dental surgeries due to a favorable combination of biocompatibility, strength and elastic modulus, and relatively low weight compared with other implantable metals [6,7,8,9]. However, the rapid degradation of Mg alloys in aqueous environments, which leads to the loss of mechanical strength and ultimately implant failure as well as an accumulation of excessive OH^−^ and H_2_, limits their clinical application [10,11]. Therefore, improving the corrosion resistance of Mg by alloying design approaches [12], microstructural modification strategies [13], and surface coating methods [14] have been demonstrated as important techniques to prolong the Mg lifetime in physiological environments [15]. Moreover, surface coatings can not only reduce and control Mg alloys’ degradation behavior but also enhance their surface biocompatibility [16,17] to ultimately accelerate tissue regeneration.

Different coatings on Mg alloys, including metal oxide [15], polymer coatings [16], inorganic nonmetallic coatings [18], and composite coatings [14], have been investigated to increase their corrosion resistance, biocompatibility, and osteointegration [19,20,21]. Among various methods, plasma electrolytic oxidation (PEO) is considered an ideal and simple technique for the surface modification of Mg alloys due to its high adhesion strength to the substrate and the adjustable composition [22,23]. PEO coatings are typically produced in modified alkaline electrolytes, such as phosphate, silicate, or sodium hydroxide solutions [24]. Depending on their thickness, microstructure, porosity, and composition, such coatings can provide an optimal combination of wear and corrosion resistance [25]. However, the formation of microspores and cracks due to PEO discharges and the limited range of composition associated with PEO coatings are the main hindrances to their applications in the biomaterial field [26]. These limitations account for the deterioration of the long-term protection and biocompatibility of PEO coatings. Therefore, the in-situ incorporation of particles into PEO coatings and the fabrication of multilayer structures are considered the best potential solutions to overcome these limitations [27].

Particle incorporation can improve various surface properties and add new functionality to a PEO coating toward the fabrication of a “smart” biomaterial [28,29]. ZnO [30], hydroxyapatite (HA) [31], and Ag particles [32] are among the most frequently studied particles that simultaneously improve the corrosion resistance and biocompatibility of the PEO surface. For instance, Liu et al. [31] investigated the effect of the incorporation of HA particles into a PEO coating and reported a significant enhancement in corrosion resistance due to the formation of a denser and more stable outer later. Because of their biocompatibility, excellent tensile strength, and high aspect ratio to inhibit crack propagation, multi-walled carbon nanotubes (MWCNTs) represent a promising potential material for improving PEO coatings for tissue engineering [33]. An important feature of carbon nanotubes is that they can incorporate drugs and corrosion inhibitors [34]. The use of MWCNT-containing electrolytes for the fabrication of PEO coatings has been previously studied, and they were shown to exhibit high corrosion and high wear resistance [35]. However, very limited information on MWCNT-containing PEO coatings on Mg alloys is available. Notably, their biofunctional features remain unknown.

An additional approach to enhance the corrosion resistance and biological properties of PEO coatings on Mg alloys is to apply polymeric and nanocomposite coatings to fill the pores resulting from PEO layer formation [36,37,38]. The degradation rate of magnesium substrates could thus be controlled [39,40]. Furthermore, these coatings can incorporate drugs and allow for their prolonged and sustained delivery [41]. Due to attributes such as biocompatibility and biodegradability, polycaprolactone (PCL) is considered a promising polymer in a wide range of biomedical applications, including bone plates, screws, and stents [42,43]. Furthermore, PCL has also been recently employed as a biomaterial for fabricating the bone scaffold for bone tissue regeneration [42,44,45]. The duplex coating with a PCL overlay on PEO with MWCNT coating can be an effective approach to improve the corrosion resistance and biological properties of Mg-based implants.

In continuation of our previous studies, in which the effects of MWCNTs and PCL on the biocorrosion and biotribology of the PEO coating were investigated [35,46], the current work is dedicated to the assessment of the potential effects of MWCNT incorporation and PCL-based duplex treatment on the biological performance of the PEO-coated AZ31B Mg alloy. Indeed, obtaining a significant improvement in biotribology by incorporating MWCNTs into the PEO coating [35] and enhancing corrosion protection by combining MWCNT incorporation and PCL duplex treatment [46] evoke the necessity for the assessment of the mentioned modification strategy on the biological response as a determining factor of the success of an implantation of a load-bearing Mg-based bone implant. The obtained results elucidate the pathway for choosing the most effective solutions and eventually reaching the desired life span of the Mg-based load-bearing implant.

## 2. Materials and Methods

### 2.1. Sample Preparation

Rectangular-shaped AZ31B Mg alloy samples with dimensions of 100 mm × 30 mm and a chemical composition (wt%) of 3.1% Al, 73% Zn, 0.25% Mn, <0.001% Cu, <0.02% Si, and 95.9% Mg were purchased from Magnesium Elektron Ltd. (Manchester, UK) and used as the base (substrate) material in this study. During preparation, the surfaces of each sample were ground using silicon carbide (SiC) abrasive papers (Struers, Copenhagen, Denmark) until achieving an average surface roughness (Sa) of 0.11 ± 0.01 μm. Subsequently, they were sequentially washed in deionized water and pure ethanol and dried with warm airflow. 

### 2.2. Plasma Electrolytic Oxidation (PEO) Process and PCL Deposition

PEO treatment was conducted as previously described [46]. In brief, PEO coating was performed in a silicate-phosphate-based solution containing 10 g/L Na_3_PO_4_·12H_2_O, 10 g/L Na_2_SiO_3_, 3 g/L CaO, and 1 g/L KOH. This electrolyte was prepared using deionized water and analytical grade chemicals at pH = 13 and an electrical conductivity of 29.7 mS/cm. The coatings were fabricated in a 2 L double jacket glass beaker. The PEO process was conducted for 600 s with a square-shaped electrical signal, a duty cycle of 50%, an initial ramp of 60 s, a frequency of 400 Hz, and a current density limit of 100 mA/cm^2^ using an EAC-S2000 power supply (ET system electronic GmbH, Altlußheim, Germany). A homemade cylindrical mesh made of 316 stainless steel was used as a counter electrode. The anodic and cathodic voltages were adjusted at +405 V and −25 V, respectively. The electrolyte was stirred during the PEO process using a magnetic stirrer (Bunsen, Madrid, Spain). The temperature of the PEO electrolyte was around 20 °C, and the electrolyte was cooled using a chiller WK1200 (Lauda, Lauda-Königshofen, Germany) during the PEO treatment.

To produce PEO coating containing MWCNT, 5 g/L MWCNTs (length: 10–30 μm, outer diameter: 20–30 nm, inner diameter: 5–10 nm, supplier US Research Nanomaterials Inc., Houston, TX, USA) were added to the electrolyte mentioned above, and the same PEO deposition conditions were applied. After the PEO process, the coated specimens were rinsed sequentially with deionized water and ethanol and dried with warm airflow.

**Figure 1:**
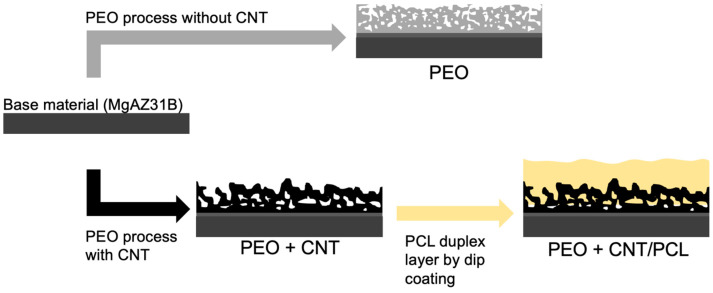
Schematic summary of the coating development procedures.

To produce the duplex coating, a polymeric (PCL) overlay was applied on top of the MWCNT-incorporated PEO coating. For this purpose, 10 g of polycaprolactone (PCL) granules (polydispersity index (PDI): 1.63, NaturePlast, Normandy, France) were dissolved in 100 mL of chloroform solvent (Analytical grade, Sigma-Aldrich, Darmstadt, Germany) under magnetic stirring for 8 h. A ND-DC 11/1 150 automatic dip coater machine (NadeTech Innovations, Pamplona, Spain) was used to deposit two PCL layers by immersing and withdrawing the specimens at a 150 mm/min speed. Coated samples were dried at room temperature for 24 h. The production of different surfaces is illustrated in Figure 1.

The following acronyms were used in this study: BM for the uncoated base material, PEO for PEO coating, PEO + CNT for PEO with incorporated MWCNTs, and PEO + CNT/PCL for the MWCNT-incorporated PEO with a PCL overlay.

### 2.3. Characterization of Base Material and Coated Surfaces

The coating thickness was measured using an eddy current meter (Fischer ISOSCOPE- FMP10, Helmut Fischer GmbH, Sindelfingen, Germany) by applying the probe randomly over the entire surface of the specimen. The cited values are an average of 10 measurements with corresponding standard deviations. The surface and cross-sectional chemistry and morphology of different coatings were investigated using a scanning electron microscope equipped with an energy dispersive spectrometer (EDS) (Philips XL30, FEI, Eindhoven, The Netherland). The average thickness was verified, and the porosity level of the coatings was assessed on the cross-section and surface of the SEM images using ImageJ software (version 1.6, National Institutes of Health, Bethesda, MD, USA). To observe the cross-sections, the specimens were first polished with a series of SiC papers with successively finer grades up to 2400 grit, followed by polishing with alumina particles (0.05 µm). Finally, the specimens were washed ultrasonically in deionized water and ethanol and dried with cold airflow.

The topography of the surfaces was evaluated by an optical profilometer InfiniteFocus SL (Alicona, Gmbh, Graz, Austria) at different magnifications. High-resolution 3D measurements (20 nm vertical resolutions with × 50 objective) were obtained. Three measurements were performed for each surface, and data are expressed as Sa (arithmetic mean height of the selected surface area)/Sz (distance between the highest peak and the deepest valley in the selected area) values.

The wettability of the uncoated and coated surfaces was assessed by determining the contact angle of a sessile droplet (2 μL) 5 min after the dropping moment using a homemade contact angle measurement device (Sharif solar Ca-500A, Tehran, Iran). The measurements were performed at the ambient temperature and in a humid chamber to minimize the evaporation of the liquid. Simulated body fluid (SBF) (8.035 g/L NaCl, 0.355 g/L NaHCO_3_, 0.225 g/L KCl, 0.231 g/L K_2_HPO_4_·3H_2_O, 0.311 g/L MgCl_2_·6H_2_O, 0.292 g/L CaCl_2_, and 0.072 Na_2_SO_4_) was used as the test liquid instead of deionized water to consider the effect of soluble salts on the surface’s wettability.

### 2.4. Biological Assays: Effects on Cell Metabolism and Cell Adhesion

For cell culture experiments, full-size rectangular-shaped specimens had to be cut into 1 × 1 cm cuboids. To facilitate the cutting process, deionized water was utilized as a lubricant. The process was carried out at room temperature. The outer edges were covered with wax to prevent the corrosion of the base material from the sides. For sterilization, samples were left overnight in 70% ethanol, dried, and exposed to UV light for 45 min on each side. BM, PEO, PEO + CNT, and PEO + CNT/PCL specimens (n = 4 per group) were incubated in cell culture media consisting of DMEM/F12 (Gibco, Thermo Fisher Scientific, Zug, Switzerland) supplemented with 10% fetal bovine serum substitute FetalClone III (HyClone, Thermo Fisher Scientific, Zug, Switzerland) and 100 U/mL penicillin + 100 mg/mL streptomycin (Sigma, Buchs, Switzerland) for 21 days. Culture media was collected after 3 h, 1, 3, 7, 14, and 21 days and frozen and kept at −20 °C until further use. TERT-immortalized human gingival fibroblasts (gFib-TERT, abm, Richmond, BC, Canada), and human bone marrow mesenchymal stem cells, bmMSCs (own cell bank), were seeded at 10,000 cells/96 wells in triplicate per time point, corresponding to the exposed media collection and material/surface type. After 48 h, the cell culture medium was removed and replaced with the collected exposed medium. After 24 h, cell metabolism was evaluated with a resazurin colorimetric assay. Optical density was read at 570 nm and 630 nm with an LEDETECT 96 microplate reader (Labexim Products, Lengau, Austria) and expressed as the ratio 530 nm/630 nm. Dimethyl sulfoxide, DMSO, 5%, (Sigma, Buchs, Switzerland), was used as a positive control to induce cell death. Cell incubation in a standard medium was used as a negative control. After the experiment, the specimens were rinsed 3 times in sterile water and air-dried.

BM, PEO, PEO + CNT, and PEO + CNT/PCL specimens previously incubated for 3 h in a cell culture medium were used for the direct cell adhesion experiment. gFib-TERT, bmMSC, and Saos-2, a human osteosarcoma cell line, were seeded at 5000 cells/cm^2^ in a cell culture medium and incubated for 24 h. After rinsing in PBS and 1 h of fixation in 10% PFA at room temperature, the specimens were rinsed in PBS and dehydrated in ascending ethanol concentrations (30, 50, 60, 70, 80, 90, 95, and 100%) for 10 min each. After drying in the ventilated hood, samples were sputtered with gold and examined under an SEM (Zeiss, Sigma 300 VP, Jena, Germany).

### 2.5. Statistical Analysis

The effect of different surfaces on cell metabolism over time was analyzed with two-way ANOVA, followed by a Bonferroni post hoc test, using Prism Version 9 for Macintosh (GraphPad Software, Inc., San Diego, CA, USA). All data are expressed as the mean ± SD. A *p*-value < 0.05 was considered statistically significant.

## 3. Results and Discussion

### 3.1. Characterisation of the Mg Alloy Surfaces

**Figure 2:**
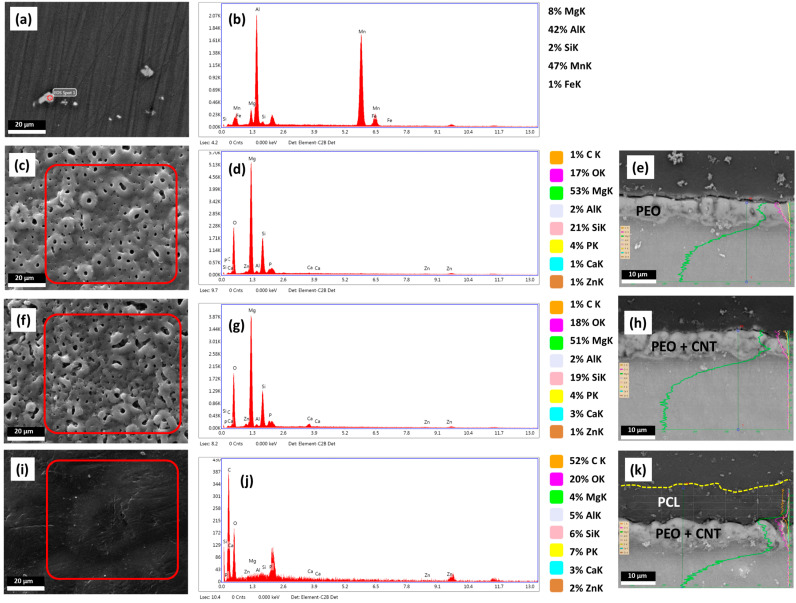
Characterization of Mg alloy surfaces. The SEM micrograph depicting the surface of BM (**a**), EDS point analysis of BM (**b**), SEM micrograph of PEO surface (**c**), EDS analysis of the selected PEO area (**d**), PEO cross-section EDS-Line (**e**), SEM micrograph PEO + CNT surface (**f**), EDS analysis of the selected PEO + CNT area (**g**), PEO + CNT cross-section EDS-Line (**h**), SEM micrograph of PEO + CNT/PCL surface (**i**), EDS analysis of the selected PEO + CNT/PCL area (**j**), PEO + CNT/PCL cross-section EDS-Line (**k**). The percentages of elements based on EDS analysis are depicted. The semi-quantitative EDS analysis results are given in %.

Surface characteristics, including the presence, distribution, and percentage of chemical elements; topography; roughness; and wettability, all influence cell responses to biomaterials [47,48]. The results of the chemical and structural analysis of the base material, namely AZ31B Mg alloy, and the three coatings are shown in Figure 2.

The presence of Al, as the main magnesium alloying element, and Mn and Fe led to the formation of intermetallic particles in the microstructure of the base material (Figure 2a). The cathodic nature of these particles can increase the degradation rate of the Mg matrix through the formation of the galvanic couples led by the high potential difference [49].

A coating could suppress the adverse effects of intermetallic particles and allow for controlled magnesium degradation rates. Three different PEO-based coatings were developed, and their surface microstructure, EDS-Map, and EDS-Line were analyzed. PEO coating without and with MWCNTs resulted in the typical creation of micro-pores, ceramic granules, and micro-cracks (Figure 2c,f). The formation of micro-pores is related to the generation of the discharge channels and gas entrapment. At the same time, micro-cracks originate from thermal stresses due to the rapid solidification of molten oxide. The porosity percentages of ~4.3% and ~15.9% were obtained for the PEO and PEO + CNT coatings, respectively, as reported previously [46], suggesting that the addition of nanoparticles leads to an increase in the irregular shape pore opening number and diameter. The EDS analysis results identified Mg, O, C, Ca, P, and Si as the major elements on the surface of all three coatings, albeit in different ratios, independent of areas or structural appearance (Figure 2d,g,j). The PEO and PEO + CNT coatings demonstrated no significant difference between their surface chemistry, with a predominance of magnesium and oxygen. The EDS-Line analyses across the deposited layers indicated that the distributions of the elements were similar for PEO and PEO + CNT. Moreover, the thickness of the PEO coating increased after the incorporation of MWCNTs (Figure 2e,h). The deposition of the PCL layer onto the PEO + CNT coating resulted in the formation of a uniform 10 μm thick polymeric layer, efficiently sealing pores and cracks (Figure 2i–k). The presence of pores was shown to improve the bonding strength between the base metal and the PCL layer [50]. The EDS-Map of the PCL coating indicated a decreased percentage of Mg and increased percentages of carbon and oxygen on the surface while detecting the other elements from the underlying PEO + CNT coating (Figure 2j), in line with previous work [50].

The uniformity and the thickness of the PCL layer are important factors in controlling magnesium degradation. Due to the dipping process constraints, the thickness of the deposited PCL layer depends on the specimen shape, particularly the length, and on the different immersion times of the specimen into the polymeric solution during each dipping cycle. Although the same conditions were employed, due to the longer specimen in the current study, the PCL layer was thinner (10 μm) than that in our previous study (60 μm) [46]. 

**Figure 3:**
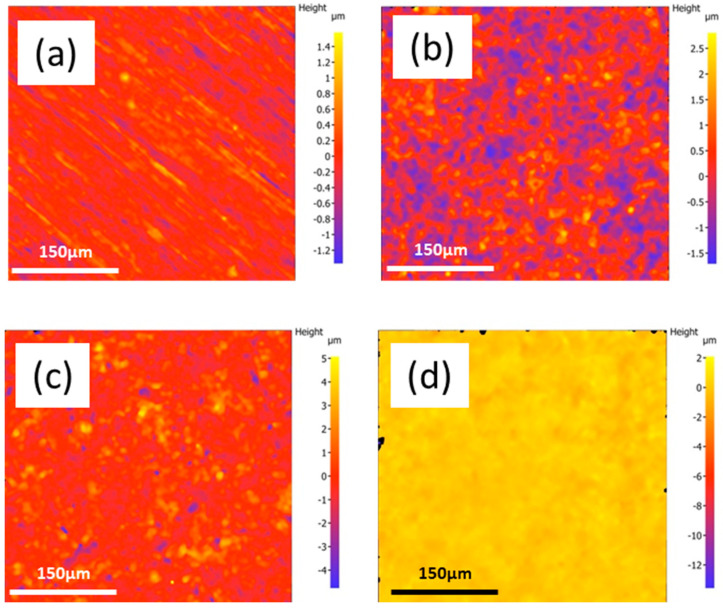
Evaluation of surface topography. Two-dimensional optical images showing the topography of the BM (**a**) PEO, (**b**) PEO + CNT (**c**), and PEO + CNT/PCL (**d**) coatings. Please note the differences in the sidebar reference scale.

The surface roughness of the coatings is one of the critical factors controlling the biological reaction to the implants [51]. In comparison with the BM roughness of Sa/Sz 0.16 ± 0.04/3.20 ± 0.15 μm, the deposition of PEO resulted in an increase of Sa/Sz to (0.46 ± 0.04)/(6.69 ± 0.62) μm, and the deposition of PEO + CNT further increased the Sa/Sz to 0.66 ± 0.08/13.32 ± 1.43 μm, respectively. These results are in agreement with the formation of larger irregular pores (openings) in the PEO + CNT coating. Applying the PLC layer to the PEO + CNT decreased the surface roughness to Sa/Sz to 0.24 ± 0.02/2.53 ± 0.15 μm. The surface roughness of the PEO coating with the PCL polymer overlay strongly decreased due to the filling of the pores and cracks. The 2D micrographs showing the topography of different coatings revealed that PEO coating resulted in an uneven surface compared with BM (Figure 3a,b), and the incorporation of MWCNTs resulted in a more pronounced uneven surface (Figure 3c). By contrast, the duplex PEO + CNT/PCL polymer coating exhibited a very smooth surface (Figure 3d).

### 3.2. Wettability

**Figure 4:**
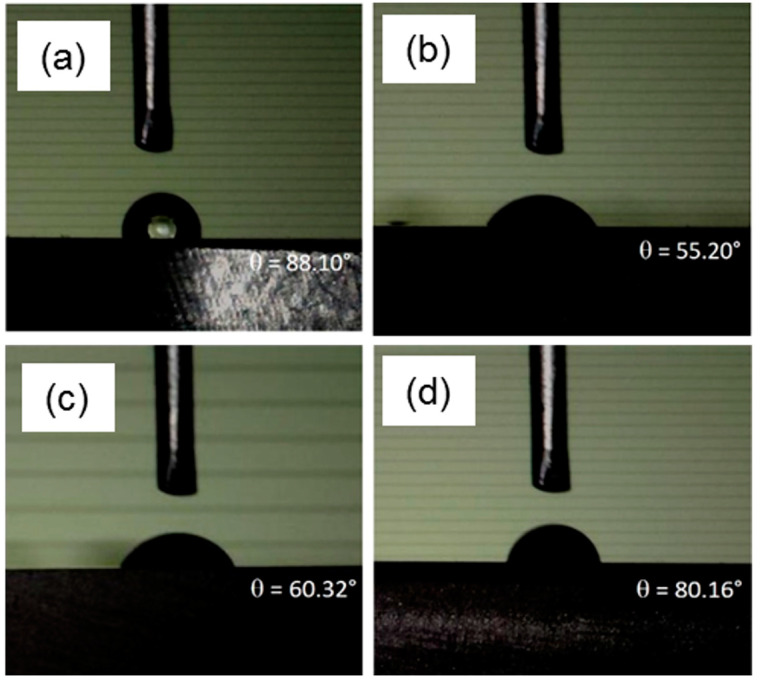
Contact angles for different magnesium surfaces. Contact angles were measured on (**a**) BM, (**b**) PEO, (**c**) PEO + CNT, and (**d**) PEO + CNT/PCL surfaces.

The wettability of the coated specimens was identified by measuring the contact angles of SBF, and the results are shown in Figure 4. Compared with the uncoated base metal, an increase in hydrophilicity, i.e., lower values of contact angles for the PEO-treated sample (~55.2°) and PEO + CNT (~60.3°) were observed, in line with previous work [50]. The hydrophobic nature of the MWCNT particles [52] could explain the slight increase in the contact angle compared with that in the PEO coating. The addition of CNT may slow down the penetration of SBF into the pores of the PEO coating. In comparison with BM, lower values for PEO + CNT/PCL specimens were observed.

### 3.3. Cell Metabolism and Cell Adhesion

Understanding the interactions between different surface properties and biological responses at the cellular level is a prerequisite for designing innovative, functional biomaterials and surface coatings. The dynamic interplay between cell materials influences cell adhesion, proliferation, and finally, differentiation [48]. Biomaterial biocompatibility relies on the cell response to the material degradation products and the surface properties the cells encounter, including wettability, roughness, surface charge, chemical functionalities, and stiffness [53,54,55,56]. Additionally, the cell type, the culturing conditions, as well as the medium composition influence cell behavior [55].

**Figure 5:**
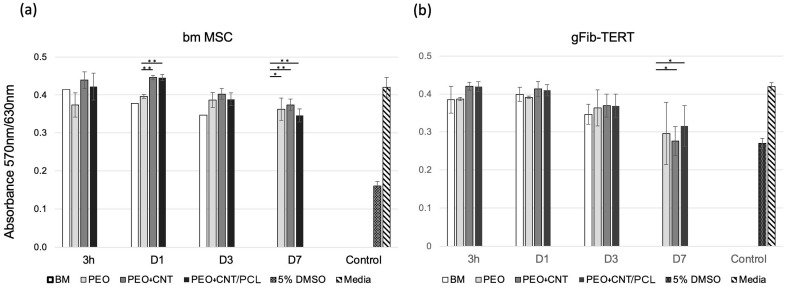
Cell metabolism on different surfaces. Bone marrow MSCs (**a**) and gFib-TERTs (**b**) metabolism expressed as an absorbance ratio measured at 570 nm and 630 nm. Cells were exposed for 24 h to the culture media collected upon incubation with BM, PEO, PEO + CNT, and PEO + CNT/PCL for 3 h, 1, 3, and 7 days. Controls comprised exposure to 5% DMSO (positive control) and fresh cell culture media (negative control). Significant differences (*p* < 0.05) are indicated: * for *p* < 0.05, and ** for *p* < 0.01.

The effect of potentially toxic agents released from the different materials/surfaces on cell metabolism was examined with two human cell types: gingival fibroblasts, gFib-TERTs, and bone marrow MSCs. In clinics, both cell types would naturally be in contact with the tested materials/surfaces. The obtained results indicated similar responses of both cell types to the media collected after incubation times from 3 h to 21 days with the studied surfaces (Figure 5a,b).

Cells were exposed to collected media for 24 h. After 3 h of incubation with media collected from BM, PEO, PEO + CNT, and PEO + CNT/PCL specimens, the metabolism of both cell types remained unperturbed and similar to that of the cells incubated in fresh cell culture media. After one day, however, while gFib-TERTs displayed similar metabolic rates independently of the surface exposure medium, there was a significantly higher metabolism in bmMSCs exposed to media collected from PEO + CNT and PEO + CNT/PCL compared with PEO. At day 3, compared with the fresh medium control, a 9% and 14% decrease in cell metabolism was observed for bmMSCs and gFib-TERTs, respectively, independent of the specimen analyzed. Exposed media collected from the BM after 7 days of incubation induced the complete death of bmMSCs and gFib-TERTs. At the same time, metabolic levels decreased by 14% for bmMSCs and 29% for gFib-TERTs, independent of the surface.

In comparison, exposure to 5% DMSO resulted in metabolic decreases of 62% and 36% for bmMSCs and gFib-TERTs, respectively, compared with fresh media. After 15 days, surface degradation and ensuing corrosion lead to complete cell death in both cell types, regardless of the coating differences (data not shown). Previous studies have demonstrated that the corrosion process can significantly change the pH of the medium and affect cell metabolism [57,58]. The pH value of human tissue is in the range of 7 ~ 7.4. Hence, a higher pH value beyond this optimal range, resulting from the release of OH- ions [59], is expected to negatively influence cell metabolism [60], in line with the presented data. DMEM is a complex solution containing salts, glucose, amino acids, and various proteins indispensable for cell survival. The precise corrosion mechanisms induced by the medium, particularly by the rapid surface adhesion of proteins and carbon, necessitate a more comprehensive analysis. The difference in the PCL layer thickness could also influence the release rate of toxic components during surface and material degradation and affect cell survival. In comparison with our previous study, the PCL layer was thinner, which could lead to its faster degradation and, consequently, the earlier exposure of cells to the corrosive environment. A thicker PCL layer should be investigated in future studies.

**Figure 6:**
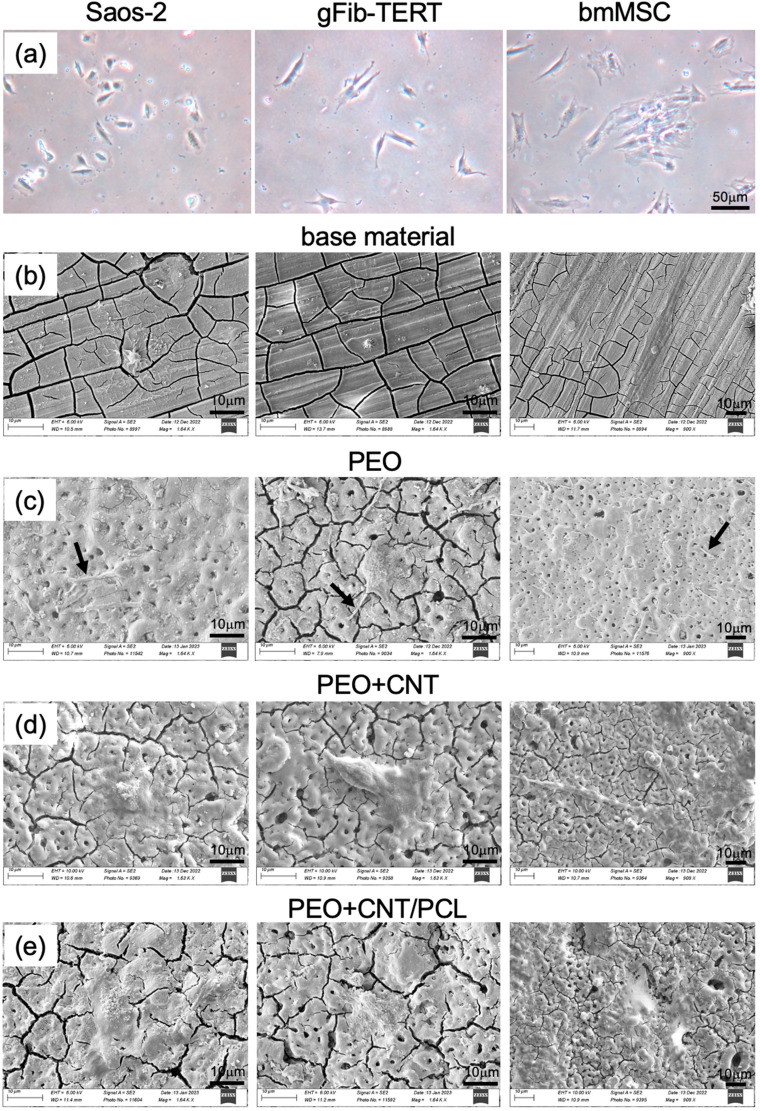
Cell adhesion and spreading on different surfaces. Phenotypic appearance of osteoblastic cell line Saos-2, oral gingival fibroblasts gFib-TERTs, and bone marrow MSCs on tissue culture plate (**a**), base material (**b**), PEO (**c**), PEO + CNT (**d**), and PEO + CNT/PCL (**e**) after 24 h. Filopodia are indicated with arrows.

Cell adhesion represents the first contact between a cell and a surface. The adhesion process comprises three stages: cell attachment to the substrate, flattening and spreading, and the formation of focal adhesion points [61]. The optimal surface for cell adhesion depends on cell type, culture conditions, surface chemistry, and topography [62,63]. The surface adhesion of three human cell types was investigated: the cell line derived from the primary osteosarcoma, Saos-2, representing osteogenic cells; bone marrow MSCs, implicated in bone regeneration; and hFib-TERTs, representing oral soft tissue cells that may encounter the investigated surfaces during the healing process. The different cell types showed different morphologies in the monolayer tissue culture: Saos-2 had a cobblestone-like appearance, bmMSCs were large and spread out, and hFib-TERTs presented a typical fibroblastic spindle shape (Figure 6a). Depending on different topographies and surface roughness, the cells displayed different adhesion characteristics. After 24 h on the base material, Saos-2 and gFib-TERTs showed signs of cell detachment and were small (Figure 6b). By contrast, bmMSCs were flat and spread on the surface, albeit with marked cell/surface boundaries and a lack of visible lamellipodia. On PEO, all three cell types were spread and produced thick cytoplasmic protrusions, filopodia, anchored into the pores (Figure 6c). Tiny pores on the order of a few microns were shown to facilitate cell attachment by providing anchoring sites [64]. A previous study demonstrated efficient adhesion of human sarcoma U2OS cells to the PEO surface [57]. A different cell response was observed with the PEO + CNT surface: the thick filopodia were not observed despite the presence of irregular openings. Instead, cells adhered to the surface but without completely spreading (Figure 6d). A previous study demonstrated a similar morphology of the Saos-2 cells on MWCNTs [65]. Such cell reaction can be due to the presence of the MWCNTs in the superficial layers of the coating. The presence of MWCNTs was shown to facilitate cell adhesion via integrin binding [66], which may be explained by the fact that their surface roughness and surface area are similar to those of collagen fibers, favoring cell attachment, proliferation, and osteogenic differentiation [67]. Additionally, Mg_2_SiO_4_, which was detected on the PEO + CNT surface, was reported to improve osteoblast adhesion [68]. Finally, the formation of very fine web-like lamellipodia with extensive and thin cell adhesion surface was observed on the PEO + CNT/PCL surface, suggesting ideal cell adhesion (Figure 6e). Similar spreading was demonstrated for the human osteoblastic cell line (MG63) on the PCL surface deposited directly onto Mg AZ31 [69].

The surface appearance of the PEO + CNT specimens used as a negative control for cell adhesion contained cracks, which contrasts our previous finding [46]. The difference could be due to the surface exposure to processes before the cell experiment, including water during specimen cutting and ethanol during sterilization. Moreover, the cutting process could have caused cracking of the PEO + CNT coating. Another potential explanation is the different composition of the solution to which the surfaces were exposed in the current study (DMEM with FCS) and a previous study (SBF).

All surfaces demonstrated hydrophilic properties, i.e., a contact angle below 90°, indicating reasonably good wettability and a potentially cell-friendly surface. For the polymer surface, optimal cell adhesion is expected in the range of 40°–70° [55]. Given the similar hydrophilicity of PEO + CNT/PCL (80°) and BM (88°), both higher than the 55° and 60° observed for PEO and PEO + CNT, respectively, a correlation between surface wettability and cell adhesion could not be established. Similarly, a comparative analysis of the effect of the hydrophilicity of different surfaces on gingival fibroblasts did not reveal a correlation [70]. Although a different solution was used for wettability assessment in this study from that in our previous study [46], leading to different contact angle values per surface, the order of the most hydrophobic to the most hydrophilic surface remained similar. Since the presence of soluble salt increases the surface tension of the SBF compared with deionized water, the contact angle values in the current study were higher. 

Surface roughness is another known parameter influencing cell adhesion. Interestingly, different cell types respond differently: while osteoblasts preferred a moderately rough surface (Sa = 1.0 to 2.0 μm) [71], a smooth surface of Sa = 0.2 μm proved optimal for human gingival fibroblast attachment and proliferation [72,73]. In this study, PEO + CNT/PCL had a lower surface roughness compared with the other surfaces and demonstrated the most efficient cell adhesion.

## 4. Conclusions

In this study, we employed a step-by-step surface modification approach to improve the biological performance of the Mg-based alloy for dental and orthopedic applications. PEO coating alone or containing MWCNTs and PEO containing MWCNTs with a duplex PCL overlayer coating were produced on the magnesium alloy AZ31B and characterized. Based on the similar metabolic cell reactions to all coated surfaces yet favorable cell adhesion on the PEO + CNT/PCL surface, the duplex PEO + CNT/PCL coating may offer a better biological functionality of the magnesium alloy. The PEO and PEO + CNT surfaces provided a platform for successful cell anchoring. The lower wettability and surface roughness of the PCL coating system compared with PEO and PEO + CNT did not affect the positive biological response of the cells. Future work should investigate the in vivo effects of these Mg alloy coatings in an animal model.

## Data Availability

The data are available upon a reasonable request to the corresponding authors.
